# Supplementary material for: SOX9 plays an essential role in myofibroblast driven hepatic granuloma integrity and parenchymal repair during schistosomiasis-induced liver damage
Source: PLoS Pathog. 2025 Jun 9;21(6):e1012928. doi: 10.1371/journal.ppat.1012928 (PMC12148231; doi:10.1371/journal.ppat.1012928)
Supplement: S1 Fig — (DOCX) [file ppat.1012928.s001.docx]

**Supplementary Figures for:**

**SOX9 expression is vital to the generation of intact granulomatous pathology during Schistosomiasis**

Kim Su, Elliot Jokl, Alice Costain, Kara Simpson, Antonn Cheeseman, Alexander Phythian-Adams, Kevin N Couper, Andrew S. Macdonald, Karen Piper Hanley


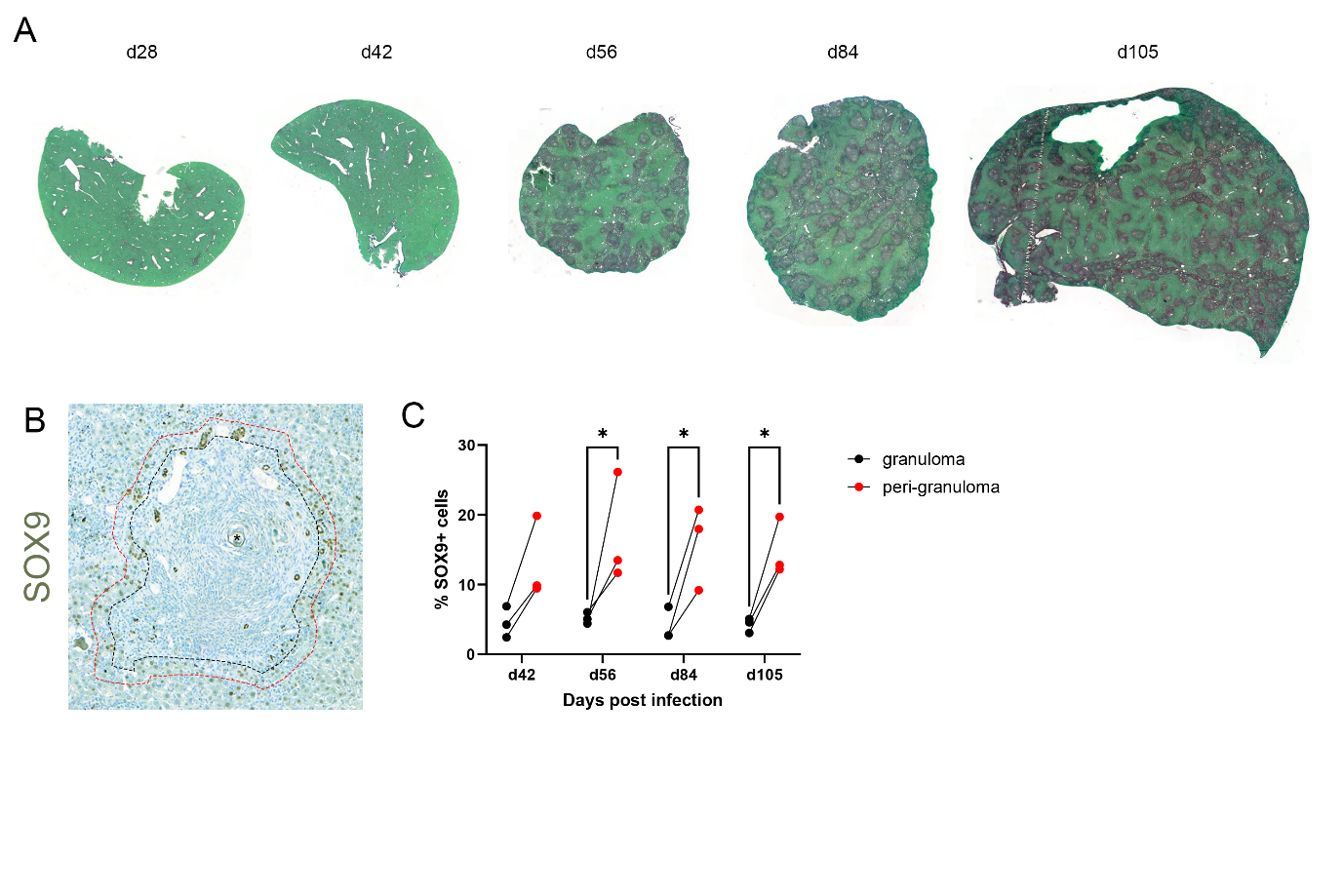


**Supplementary figure 1 – Lobe-level view of fibrotic granuloma formation during infection timecourse and comparison of granuloma and peri-granuloma levels of SOX9**

**A -** PSR staining of liver after 28, 42, 56, 84 and 105 days respectively shows the progressive formation of fibrotic granulomas during infection.

**B** - Representative image of SOX9 staining. The black dotted line represents the outline of the granuloma. The red dotted line represents the peri-granuloma region (arbitrarily a 50µm border around the granuloma edge)

**C** - Quantification of percentage of SOX9+ cells in the granuloma (black) and peri-granuloma (red) regions at the indicated time point. Each point represents an average of three regions per animal. N=3 animals per time point. *=p<0.05, 2-way ANOVA with Sidak’s multiple comparisons test.
