# Supplementary material for: SOX9 plays an essential role in myofibroblast driven hepatic granuloma integrity and parenchymal repair during schistosomiasis-induced liver damage
Source: PLoS Pathog. 2025 Jun 9;21(6):e1012928. doi: 10.1371/journal.ppat.1012928 (PMC12148231; doi:10.1371/journal.ppat.1012928)
Supplement: S2 Fig — (DOCX) [file ppat.1012928.s002.docx]

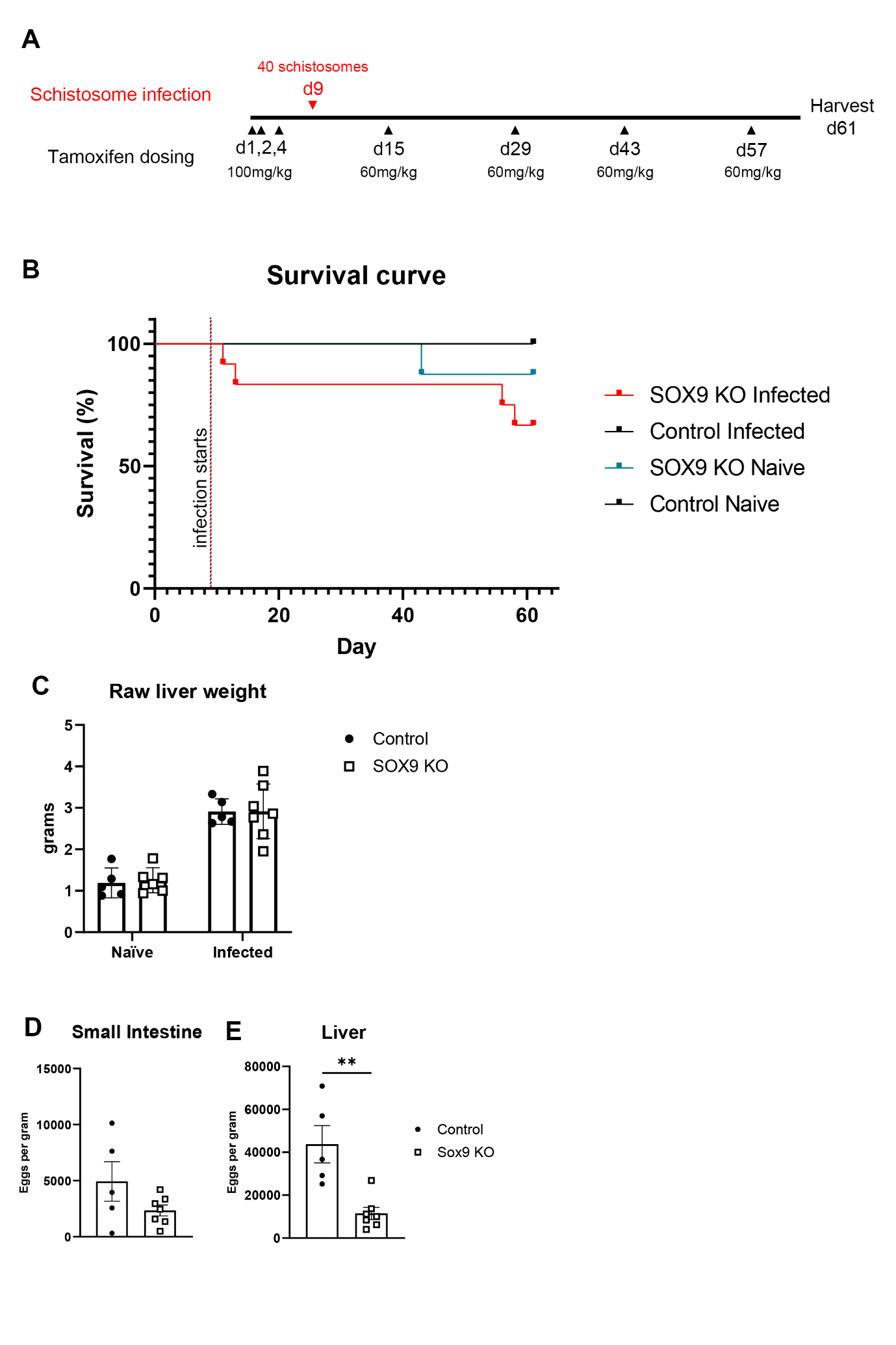


**Supplementary figure 2 – Experimental time course and survival curve**

**A** – Overview of the experimental plan and treatment timings

**B** - Percentage survival of each group over time from the first tamoxifen injection. Infection was performed on experimental day 9. Note that control infected and naive groups had 100% survival and thus overlapping survival curves.

**C –** Raw liver weights of animals uncorrected for body weight

**D** - Egg count per gram in the small intestine

**E** – Egg count per gram in the liver. Student’s T-test, **=p<0.01
