# Supplementary material for: SOX9 plays an essential role in myofibroblast driven hepatic granuloma integrity and parenchymal repair during schistosomiasis-induced liver damage
Source: PLoS Pathog. 2025 Jun 9;21(6):e1012928. doi: 10.1371/journal.ppat.1012928 (PMC12148231; doi:10.1371/journal.ppat.1012928)
Supplement: S3 Fig — (DOCX) [file ppat.1012928.s003.docx]

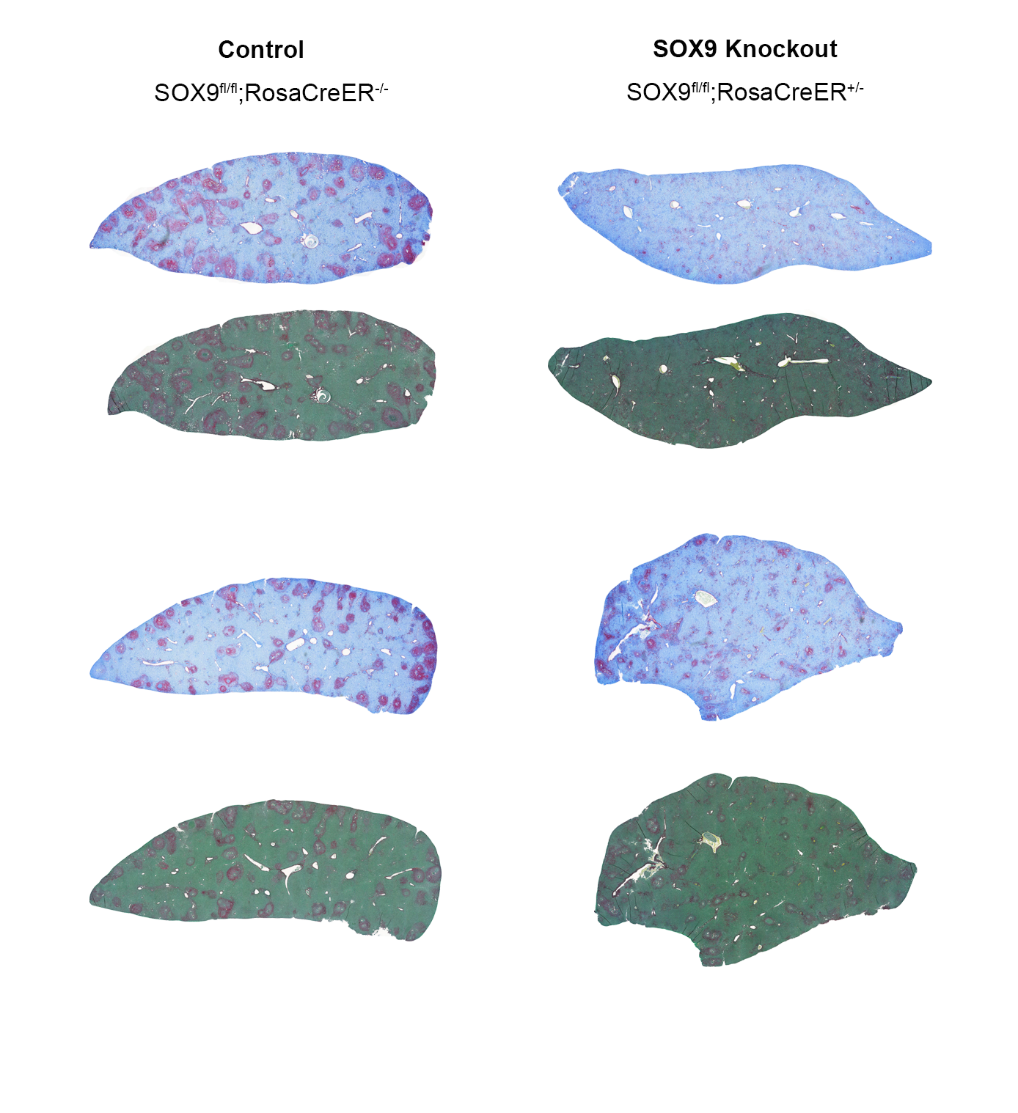


**Supplementary Figure 3 Representative Lobe level view of PSR and aSMA staining**

Representative lobe level views of PSR and aSMA IHC in (left) control and (right) SOX9 KO infected animals. Control infected mice show discrete granuloma fibrosis while KO infected mice show more diffuse patterning.
