# Supplementary material for: SOX9 plays an essential role in myofibroblast driven hepatic granuloma integrity and parenchymal repair during schistosomiasis-induced liver damage
Source: PLoS Pathog. 2025 Jun 9;21(6):e1012928. doi: 10.1371/journal.ppat.1012928 (PMC12148231; doi:10.1371/journal.ppat.1012928)
Supplement: S4 Fig — (DOCX) [file ppat.1012928.s004.docx]

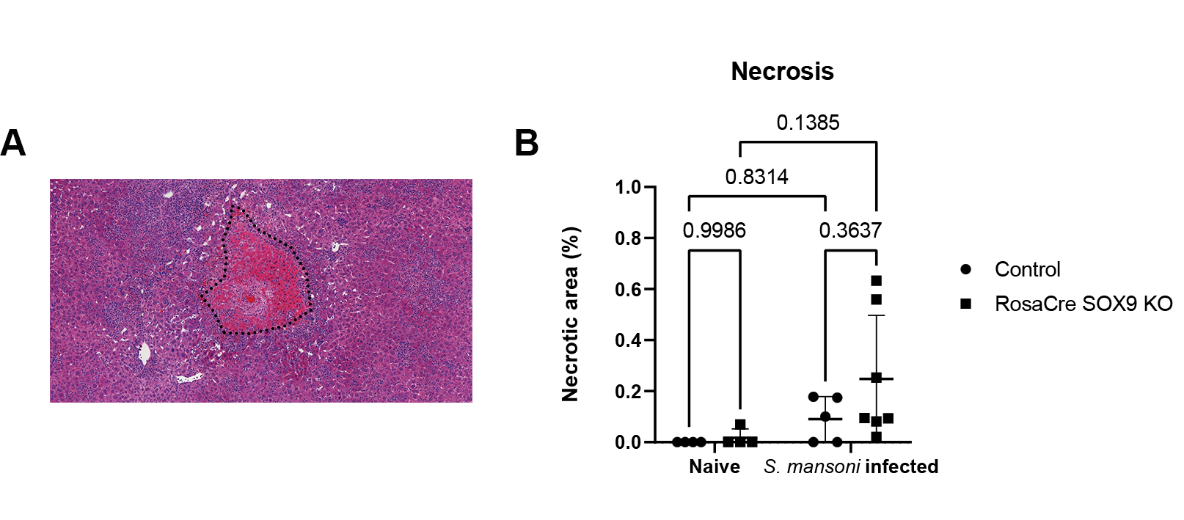


**Supplementary figure 4 – Quantification of necrosis**

**A** Representative image of a region of necrosis (black dotted border)

**B** Quantification of necrotic area as a percentage of total lobe area across treatment groups. Statistical analysis performed using 2-Way ANOVA.
