# Supplementary material for: SOX9 plays an essential role in myofibroblast driven hepatic granuloma integrity and parenchymal repair during schistosomiasis-induced liver damage
Source: PLoS Pathog. 2025 Jun 9;21(6):e1012928. doi: 10.1371/journal.ppat.1012928 (PMC12148231; doi:10.1371/journal.ppat.1012928)
Supplement: S5 Fig — (DOCX) [file ppat.1012928.s005.docx]

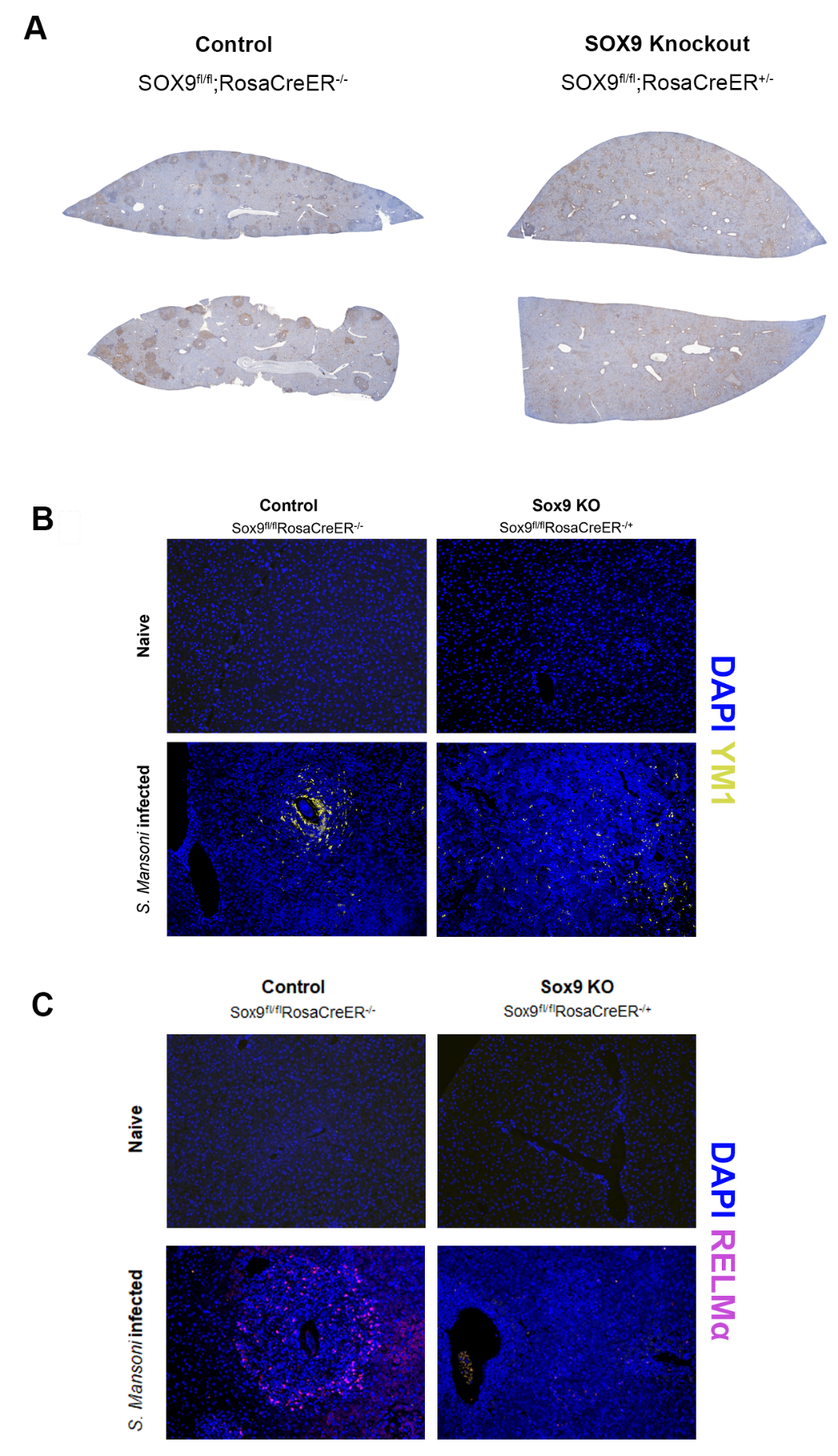


**Supplementary figure 5 – Immune cell distribution in infected control and SOX9 KO animals**

**A -** Representative lobe level views of IHC against F4/80 in (left) control and (right) SOX9 knockout animals. Control animals show mainly discrete granuloma patterning whereas knockout animals show more diffuse patterning.

**B** - Representative immunofluorescence images of hepatic tissue stained for Ym1 (yellow). Data are from 2 separate experiments. n= 4-7 mice per group

**C -** Representative immunofluorescence images of hepatic tissue stained for Relma (pink). Data taken from a single experiment
