# Supplementary material for: SOX9 plays an essential role in myofibroblast driven hepatic granuloma integrity and parenchymal repair during schistosomiasis-induced liver damage
Source: PLoS Pathog. 2025 Jun 9;21(6):e1012928. doi: 10.1371/journal.ppat.1012928 (PMC12148231; doi:10.1371/journal.ppat.1012928)
Supplement: S6 Fig — (DOCX) [file ppat.1012928.s006.docx]

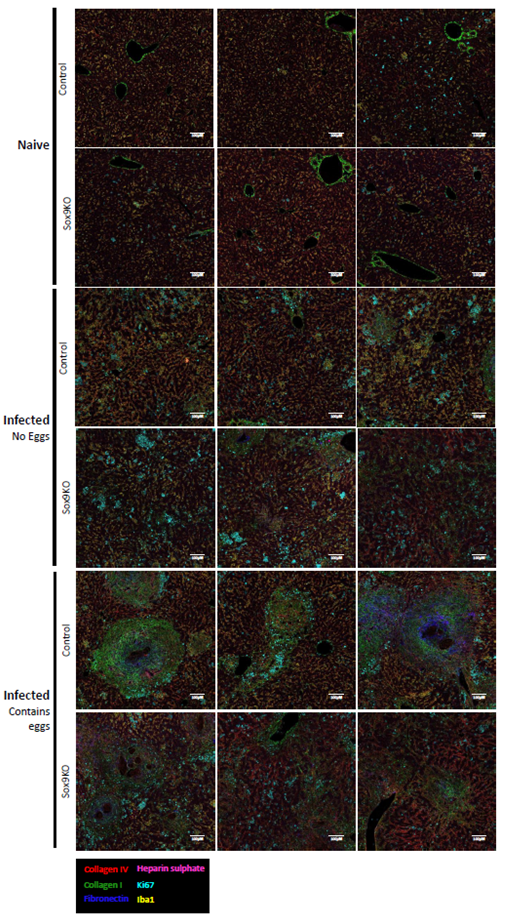


**Supplementary Figure 6 – Extended Hyperion data shows fragmented ECM deposition in *Sox9* deficient animals**

Representative Hyperion imaging mass cytometer images taken from the liver of naïve WT (n=2) and KO (n=2) and *S. mansoni* infected WT (n=2) and KO (n=2) mice. Images taken from egg-containing or egg-free zones. Staining for the indicated markers in the colours shown.
