# Supplementary material for: SOX9 plays an essential role in myofibroblast driven hepatic granuloma integrity and parenchymal repair during schistosomiasis-induced liver damage
Source: PLoS Pathog. 2025 Jun 9;21(6):e1012928. doi: 10.1371/journal.ppat.1012928 (PMC12148231; doi:10.1371/journal.ppat.1012928)
Supplement: S7 Fig — (DOCX) [file ppat.1012928.s007.docx]

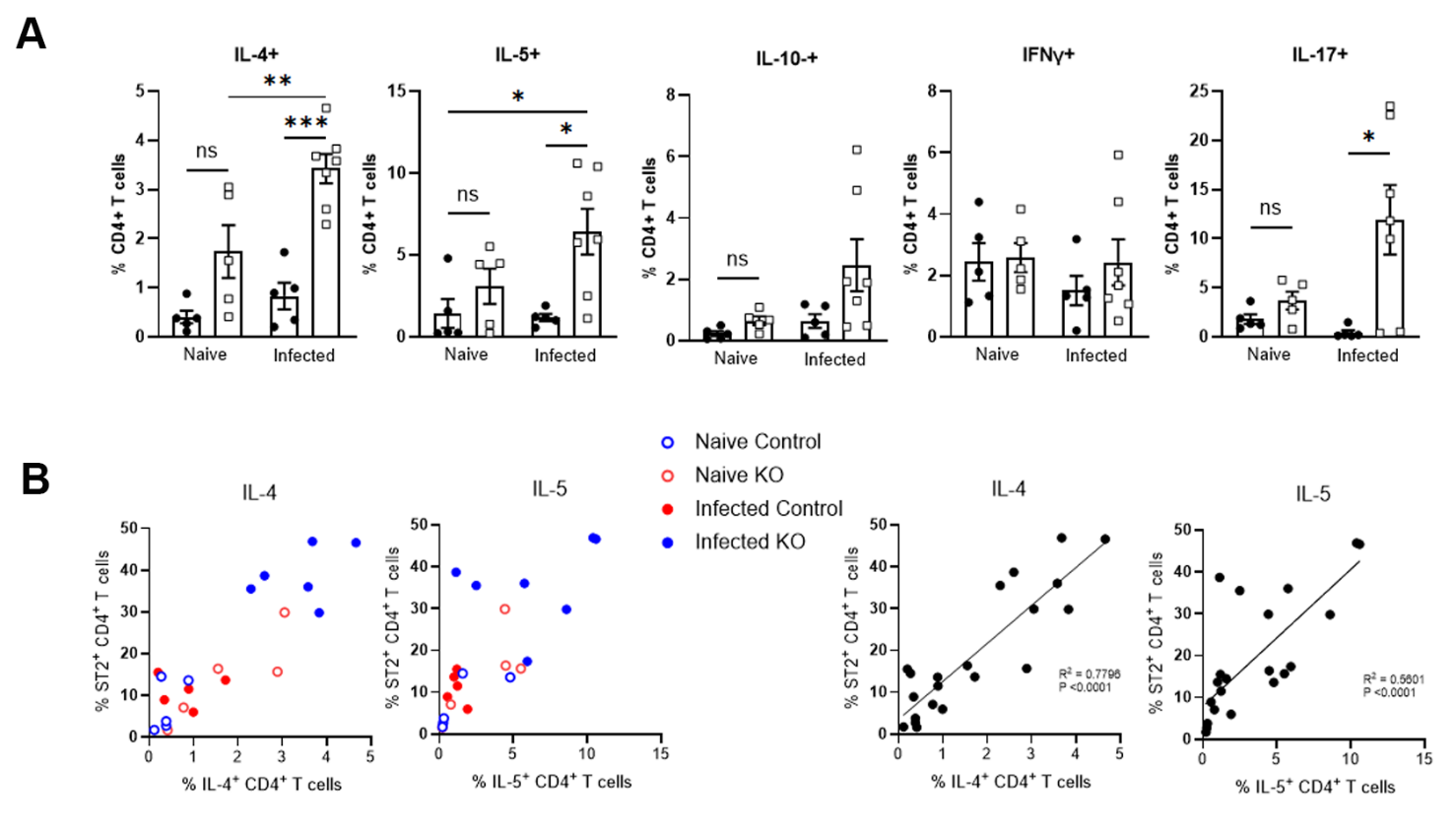


**Supplementary Figure 7 – Additional immune characterisation**

**A** - Cytokine secretion from PMA/Ionomycin stimulated hepatic CD4+ T cells. Data shown as %CD4+ T cells positive for respective intracellular cytokine. n=5-7 per group, pooled from 2 separate experiments. Statistical significance determined by one-way ANOVA and Dunnett’s multiple comparisons test (*p<0.05, **p<0.01, ***p<0.001, ****p<0.0001).

**B** - Correlation between ST2 expression on CD4+ T cells and expression of intracellular cytokines IL-4 and IL-5. A) Data points coloured according to experimental groups B) All data points grouped an analysed by Simple Linear Regression analysis (*p<0.05, **p<0.01, ***p<0.001, ****p<0.0001).
