# Supplementary material for: SOX9 plays an essential role in myofibroblast driven hepatic granuloma integrity and parenchymal repair during schistosomiasis-induced liver damage
Source: PLoS Pathog. 2025 Jun 9;21(6):e1012928. doi: 10.1371/journal.ppat.1012928 (PMC12148231; doi:10.1371/journal.ppat.1012928)
Supplement: S1 Table — (DOCX) [file ppat.1012928.s008.docx]

**Supplementary Table 1 – Antibodies used for Flow Cytometry**

| **ANTIBODY** | **COMPANY** | **CLONE** | **Used for** |
| --- | --- | --- | --- |
| Ly6C | Biolegend | HK1.4 | Myeloid gating/ panel |
| CD64 | Biolegend | x54-5/7.1 | Myeloid gating/ panel |
| XCR1 | Biolegend | ZET | Myeloid gating/ panel |
| CD11c | Biolegend | N418 | Myeloid gating/ panel |
| pDCA-1 | Biolegend | 927 | Myeloid gating/ panel |
| CD11b | Biolegend | M1/70 | Myeloid gating/ panel |
| CD45 | Biolegend | 30-F11 | Myeloid gating/ panel |
| Zombie UV | Invitrogen | N/A | All panels |
| Siglec-F | BD | E50-2440 | Myeloid gating/ panel |
| MHC-II | eBioscience | MS/114.15.2 | Myeloid gating/ panel |
| F4/80 | Biolegend | BM8 | Myeloid gating/ panel |
| CD3 | Invitrogen | 17A2 | Myeloid gating/ panel (lineage) |
| CD19 | Invitrogen | Ebio(ID3) | Myeloid gating/ panel (lineage) |
| NK1.1 | Invitrogen | PK136 | Myeloid gating/ panel (lineage) |
| Ly6G | Biolegend | 1A8 | Myeloid gating/ panel |
| Ter119 | eBioscience | Ter119 | Myeloid gating/ panel (lineage) |
| CD49b | eBioscience | DX5 | Myeloid gating/ panel (lineage) |
| CD4 | Biolegend | RM4-5 | T cell and cytokine gating/ panel |
| TCRβ | Invitrogen | H57-597 | T cell and cytokine gating/ panel |
| *CD45* | Biolegend | 30-f11 | T cell and cytokine gating/ panel |
| CD25 | Biolegend | PC61 | T cell and cytokine gating/ panel |
| Rorγt | Ebioscience | B2D | T cell and cytokine gating/ panel |
| IL-10 | Biolegend | JES5-16E3 | Cytokine gating/ panel |
| IL-17 | Biolegend | TC11-1810.1 | Cytokine gating/ panel |
| IL-4 | Biolegend | 11B11 | Cytokine gating/ panel |
| IL-5 | Ebioscience | TRFK.5 | Cytokine gating/ panel |
| IFNy | Biolegend | XMG1.2 | Cytokine gating/ panel |
