# Supplementary material for: SOX9 plays an essential role in myofibroblast driven hepatic granuloma integrity and parenchymal repair during schistosomiasis-induced liver damage
Source: PLoS Pathog. 2025 Jun 9;21(6):e1012928. doi: 10.1371/journal.ppat.1012928 (PMC12148231; doi:10.1371/journal.ppat.1012928)
Supplement: S2 Table — (DOCX) [file ppat.1012928.s009.docx]

**Supplementary Table 2 – Antibodies used for Hyperion image acquisition**

| Metal | Target | Clone | Supplier |
| --- | --- | --- | --- |
| In115 | B220 | RA3-6B2 | BioLegend |
| La139 | Pan-cytokeratin | AE-1/AE-3 | BioLegend |
| Nd143 | Vimentin | RV202 | Abcam |
| Sm147 | Fibronectin | EPR23110-46 | Abcam |
| Nd150 | CD44 | IM7 | BioLegend |
| Sm152 | Pan-laminin | Polyclonal, rabbit IgG | ThermoFisher |
| Gd156 | F480-PE/*anti-PE* | BM8/*PE001* | BioLegend/*BioLegend* |
| Gd158 | FoxP3 | FJK/16s | ThermoFisher |
| Gd160 | Collagen IV | Polyclonal, rabbit IgG | Abcam |
| Dy161 | Iba1 | Polyclonal, rabbit IgG | WAKO |
| Dy162 | VersicanV0V1 | Polyclonal, rabbit IgG | ThermoFisher |
| Dy163 | PTEN | 6H2.1 | Merck |
| Er168 | Ki-67 | B56 | BD Biosciences |
| Tm169 | Collagen I | Polyclonal, rabbit IgG | Standard BioTools |
| Yb174 | MHCII (I-A/I-E) | M5/114.15.2 | Standard BioTools |
| Lu176 | Syndecan 1-APC/*anti-APC* | 281-2/*APC003* | BioLegend/*Standard BioTools* |
| Pt196 | Heparan sulfate | F58-10E4 | Amsbio |
