# Supplementary material for: SOX9 plays an essential role in myofibroblast driven hepatic granuloma integrity and parenchymal repair during schistosomiasis-induced liver damage
Source: PLoS Pathog. 2025 Jun 9;21(6):e1012928. doi: 10.1371/journal.ppat.1012928 (PMC12148231; doi:10.1371/journal.ppat.1012928)
Supplement: Attachment — Submitted filename: Covering letter & rebuttal Final.docx [file ppat.1012928.s012.docx]

Professor Karen Piper Hanley

**Manchester Cell-Matrix Centre**

**Division of Diabetes, Endocrinology & Gastroenterology**

Faculty of Biology, Medicine & Health

University of Manchester, UK.

Karen.piperhanley@manchester.ac.uk

**Wellcome Trust Centre for Cell-Matrix Research**

Faculty of Biology, Medicine & Health

University of Manchester

Karen.piperhanley@manchester.ac.uk

2^nd^ April, 2025

Dear Editor,

*Re: Research article re-submission:* ***PPATHOGENS-D-25-00178 ‘*SOX9 plays an essential role in myofibroblast driven hepatic granuloma integrity and parenchymal repair during schistosomiasis-induced liver damage*’*** *led by Dr Karen Piper Hanley*

We thank the reviewers for their helpful comments and the editor for handling this manuscript. We have provided a full rebuttal to all comments below.

We thank all three reviewers for their support and thoughtful assessments of our manuscript.

**We would initially like to highlight an error in our initial submission**. We presented an incorrect version of Figure 4 where we had modelled the effect of pooling failed infections with naïve mice. However, our statistical analyses and conclusions as presented in the paper corresponded to analyses with those mice rightly excluded, as even a failed infection could ostensibly disturb the immune environment. We have replaced this figure with the correct version in our resubmission. No conclusions of the paper are impacted by this error. We apologise for this oversight.

We provide below in red our responses to suggested modifications from Reviewer #2 in the “Minor Issues” section.

Fig. 1. Can levels of SOX9 within granulomas vs adjacent to granulomas be quantified? Figure legend states SOX9 localises to areas of SMA and PSR stain, but it appears much of the brown SOX9 stain is adjacent to the granuloma (if delineated by PSR staining on what appear to be sequential sections). This is also be case in Fig. 2A where intense SOX9 red stain is adjacent to what might be an egg (?). It makes sense if SOX9 is adjacent to but not within the granulomas given expression by damaged hepatocytes rather than the immune infiltrate.

We thank the reviewer for this thoughtful observation. We acknowledge that the original wording in the Figure 1 legend may suggest an exclusive localisation to SMA-expressing regions, which we later clarify in Figure 2 as one of several regions/cell types in which SOX9 is expressed during injury. The reviewer is correct to identify, as we show in Figure 2C, that e.g. injured hepatocytes surrounding the granuloma also express SOX9. In line with these suggestions, we have quantified SOX9 levels both within and adjacent to granuloma regions across the WT time-course of infection (Supp. Figure 1B&C) and modified the legend for figure 1 to more accurately reflect SOX9 localisation.

Fig. 2B white * for egg referred to in legend is missing?
We have added this, thank you.

- Supp Fig. 2B. There are perhaps 2x very early deaths in the SOX9-/- mice. Is there any evidence that lung inflammatory responses to migrating larvae are exaggerated in SOX9-/- mice? The additional wave of deaths ~wks6-7 is consistent with egg-induced pathology and convincing given the granuloma changes shown. I also wondered if there were any changes in intestinal inflammation/fibrosis due to egg migration, but this is perhaps more beyond scope of study.

We appreciate the reviewer’s insightful interpretation of survival and the suggestion regarding potential early pulmonary inflammation in SOX9-/- mice. While the early deaths could indeed reflect an enhanced inflammatory response to migrating larvae, our study was specifically designed to investigate liver pathology, and we did not assess lung or intestinal tissues in this context.

We agree that exploring lung or intestinal inflammatory responses in SOX9-/- mice could be highly informative, particularly in relation to early mortality and broader systemic responses to infection. However, these questions lie outside the current scope and dataset of this study. We hope to address these avenues in future work.

- Fig. 3E. Representative Western does not really back up the graphed data i.e. wide variation in (stain-free) total protein levels between different samples. Assuming this is effectively a loading control, are COL1 levels corrected for this in the graph? There are only 2x naïve and 2x infected SOX9ko samples for the Western with big difference in total protein levels within the (small) groups. Given there are 5-7 infected mouse datapoints on the graph, it would be good to have more convincing representative blots.

We thank the reviewer for this careful observation. As noted, the stain-free total protein signal serves as a loading control, and all COL1 expression levels in the graph are normalized to this signal to account for any loading variation. We agree that the originally shown representative blot did not fully capture the consistency of the full dataset, particularly given the limited number of samples shown. To better reflect the underlying data, we have now included additional representative blots from the broader cohort, which more clearly support the quantification presented in the graph. These updated blots are included in the revised Figure 3E.

- Page 7, “trend towards increased necrosis in the SOX9-/- infected group, but this was not significant”. Given p=0.36 and only 2/7 mice show elevated necrosis, this is a bit of a stretch. I suggest either repeating infection experiment or removing this statement.

We appreciate the reviewer’s critical reading of this statement. Given the lack of statistical significance (p=0.36) and the limited number of affected animals, we agree that the description of a "trend" is not sufficiently supported. While necrosis remains a relevant pathological feature in schistosomiasis, we have revised the text to more accurately reflect the data and now state simply that *“no significant differences were observed in levels of necrosis.”* We believe this provides a more accurate and appropriately conservative interpretation of the findings.

- Were circulating liver enzymes (e.g. AST/ALT) measured? This would support increased liver damage in KO mice.

We appreciate this suggestion. Unfortunately, we did not measure serum-based markers as part of this study. Our focus was primarily reflected in the histopathological and molecular analyses of liver tissue, including fibrosis and cellular responses.

- Fig. 4. Do you have liver cell number data (total cell counts and for individual cell populations)? This would answer whether eosinophils are genuinely elevated in the KO mice, or whether another cell type (macrophages?) are reduced, and so eosinophils become proportionally greater.

Does the increase in CD25(+) T cells reflect stronger T cell activation (potentially not, given ICC data) or more Tregs? This could be answered with Foxp3 staining.

We thank the reviewer for these thoughtful and helpful suggestions. We agree that absolute cell count data would add valuable context to the flow cytometry findings. However, in this study, only a single liver lobe was processed per animal rather than the entire liver, and due to an oversight, individual lobe weights were not recorded at the time of tissue processing. Consequently, we are unable to calculate total liver cell numbers per sample, as variation in lobe size and cellularity may have introduced confounding factors. To partially address this, we have included total liver weights for each group in **Supplementary Figure 2C**. These show no significant differences between control and KO-infected mice, suggesting that total liver cellularity is broadly similar. While this allows for a cautious inference that eosinophil numbers may be increased in SOX9-/- mice, we agree that without direct cell count data, this cannot be stated conclusively in the manuscript.

Regarding CD25(+) T cells, we appreciate the reviewer’s point about distinguishing between activated T cells and regulatory T cells. While Foxp3 was included in our staining panel, results were inconsistent across experiments, Given this variability, we opted not to include the Foxp3 data in the manuscript, as we could not confidently interpret its significance. However, we agree that this is an important line of inquiry and plan to pursue it in future studies with expanded T cell phenotyping.

- What is the justification for presenting cytokine levels relative to respective WT or KO control groups? Were there any differences between WT and KO naïve levels? And was this because the results are pooled from multiple experiments with different cytokine levels across harvests?

Thank you for your comment. Since the data were pooled from two separate experiments and there was inherent variability between animals, we presented the results as fold change. For all cytokines analysed, we did not observe any differences between naïve control and naïve KO mice (added as **Supplementary Figure 7A**).

Notably, when cytokine values are presented as raw values rather than fold change, more statistical differences emerge between infected and naïve groups. However, given the variability between mice and experiments, we believe presenting the data as fold change provides a fairer comparison.

- Was ST2 and intracellular cytokine staining carried out on the same cells? It would be good to correlate e.g. type 2 cytokines with IL-33 receptor expression if possible.

Unfortunately, we used two separate T cell-focused panels: (i) T cell transcription factors and mediators, and (ii) intracellular cytokine secretion. ST2 was only included in the first panel, meaning we cannot determine which specific cell type produces type 2 cytokines.

As you suggested, we explored potential correlations between ST2 and IL-4/IL-5 (added as **Supplementary Figure 7B**). However, given the numerous mediators, signalling pathways, and other factors that increase during infection, we cannot conclude from these correlations that ST2 signalling directly contributes to the rise in these cytokines.

- Supp Table 1 gives details on several Ab that are not included in the study (including Foxp3 as mentioned above). Do you have data on RELMα expression in liver macrophages and eosinophils? It would be good to see how this compares to liver Ym-1 (with dual stain for F4/80 or other macrophage markers). This is also interesting given elevated % eosinophils in KO liver (are they more activated as well as more prevalent?). Similarly, iNOS staining was carried out according to Supp Table so does this show if KO liver macrophages adopt any M1 bias? If data is not included/was not carried out then please amend table.

Apologies - the supplementary data tables list all the antibodies that were used in our immune panel. Unfortunately, several of these did not work well in our mouse tissue. We have amended the table to reflect this.

We agree that data on RELM-α and iNOS would have been a valuable addition to the paper. We did attempt to assess RELM-α, but it was only detectable by immunofluorescence in the first experiment (shown now in Supplementary 5C). iNOS was included in our flow cytometry panel, but the results were inconclusive. As such we are unable to comment on the M1 bias in this current work.

- Supp Fig. 5B. I am not sure if I am misreading but is the data in correct order? i.e. top right naïve KO appears to have an egg surrounded by Ym-1(+) cells, so this could be WT infected? In addition, it would be good to combine Ym1 stain with F4/80 to determine if macrophages, as per point above (i.e. are the Ym1+ cells macrophages?)

We thank the reviewer for spotting this mistake. The top right and bottom left images were accidentally swapped, but we have now corrected them in the supplementary figure. Unfortunately, co-staining with Ym1 and F4/80 antibodies isn’t possible due to host incompatibilities. However, we agree it would be an interesting approach for future studies.

- Page 13 M/M indicates that egg counts in liver and intestine were performed. Please include this data.

We apologise for mistakenly omitting this data from the manuscript. It has now been included as part of Supplementary Figure 2 D and E. While small intestinal egg counts remain unchanged between control and KO mice, liver egg counts are significantly lower in KO mice.

To accompany this data, we have added the following text: *"**Interestingly, KO mice showed a significant reduction in hepatic, but not small intestinal egg burden compared to controls. This may reflect differences in liver architecture, with eggs being redirected to extrahepatic sites due to impaired ECM deposition and fibrosis."*

- Fig. 5 Hyperion data is very impressive. Is there any data to identify the proliferating (Ki67+) cells that are mostly on the edges of the granuloma in WT mice? Additionally, can proliferating cells be quantified to show whether their numbers as well as spatial organisation changes in KO mice?

Thank you. Unfortunately, our Hyperion panel did not include antibodies that would assist in identifying these cells. We assume based on other histology data and nuclear morphology that the proliferating cells around the granuloma are likely predominantly hepatocytes with some cholangiocytes from proliferating ducts in response to injury, consistent with our observations in Figure 2.

We have attempted to examine for differences in Ki67 quantification based on the Hyperion data. Since the n=2 animals per group is not sufficiently powered, we treated each region as a pseudo-replicate. As per the graph below, though the elevated proliferation between naïve and infected animals was clear, we see no evidence of a genotype effect. Since the data only represents an actual n=2, we would propose not to include this in the manuscript but provide the analysis for the benefit of the reviewers:

In terms of a spatial phenotype, there is a striking localisation of ki67 to the granuloma edge in WT samples that is less apparent in KO, presumably because of the lack of granuloma organisation. However, there is also a high background of Ki67 expression throughout the tissue in both genotypes and thus we have not been able to capture a clear quantitative organisational phenotype.

- Page 11, “infected SOX9-/- mice display an enhanced potential to produce the immunoregulatory cytokine IL-10” is not case in Fig. 4D.

The reviewer is correct that our observed trend is not significant. This was an oversight from a previous version of the manuscript. We have removed this statement from the discussion.

- Details on intracellular cytokine staining (PMA, ionomycin, brefeldin etc) missing from M/M.

Thank you for bringing this to our attention. We have included the following text in the materials and methods section of the manuscript: ‘*For intracellular cytokine staining, cells were plated at 0.4-1 × 10^6^ cells /well in volumes of 200μl and stimulated for 3h in the presence of 30ng/ml PMA, 1 µg/ml Ionomycin and 1 µg/ml Golg Stop (BD), before processing for flow cytometry’*

Thank you for considering our work.

Kind Regards,

Karen Piper Hanley

Professor of Molecular Medicine

Head of Division in Diabetes, Endocrinology and Gastroenterology
